# Increased expression of survivin in gastric cancer patients and in first degree relatives

**DOI:** 10.1038/sj.bjc.6600421

**Published:** 2002-07-15

**Authors:** J Yu, W K Leung, M P A Ebert, E K W Ng, M Y Y Go, H B Wang, S C S Chung, P Malfertheiner, J J Y Sung

**Affiliations:** Department of Medicine and Therapeutics, Prince of Wales Hospital, Chinese University of Hong Kong, Shatin, Hong Kong; Department of Surgery, Prince of Wales Hospital, Chinese University of Hong Kong, Shatin, Hong Kong; Department of Gastroenterology, Hepatology, and Infectious Diseases, Otto-von-Guericke University, Magdeburg, Germany

**Keywords:** gene expression, survivin, gastric cancer, cyclo-oxygenase-2

## Abstract

Survivin was recently described as an apoptosis inhibitor. Its pathogenic role in gastric cancer is largely unknown. Expression of survivin in gastric cancer and non-cancer first-degree relatives, and its association with apoptosis and cyclo-oxygenase-2 expression was investigated. Fifty gastric cancer, 30 non-cancer first-degree relatives, 20 normal controls and five gastric cancer cell lines were studied. Survivin and cyclo-oxygenase-2 were evaluated by reverse transcriptase-polymerase chain reaction, immunohistochemistry and Western blot. Survivin expression was absent from normal gastric mucosa. All five cancer cell lines and 34 out of 50 (68%) human gastric cancer tissues expressed survivin mRNA. Survivin expression was less frequent (22%; *P*<0.001) in adjacent non-tumour gastric tissues. Immunohistochemistry and Western blot obtained similar findings. Gastric cancers with survivin expression displayed significantly reduced apoptosis (*P*=0.02), and associated with cyclo-oxygenase-2 overexpression at both mRNA (*P*=0.001) and protein levels (*P*=0.041). Moreover, survivin mRNA was detected in the gastric mucosa of eight (27%) non-cancer relatives. Expression in non-cancer patients showed positive correlation with *H. pylori* infection (*P*=0.004). This demonstrates the frequent expression of survivin in gastric cancer and in first-degree relatives. Co-expression of survivin and cyclo-oxygenase-2 may suggest multiple pathways contributing to the inhibition of apoptosis in gastric cancer.

*British Journal of Cancer* (2002) **87**, 91–97. doi:10.1038/sj.bjc.6600421 www.bjcancer.com

© 2002 Cancer Research UK

## 

Regulation of apoptosis, or programmed cell death, is crucial to the preservation of homeostasis and morphogenesis of human tissue (Vaux *et al*, 1994). Disturbance of this process by aberrantly extending cell viability or favouring accumulation of transforming mutation is thought to contribute to carcinogenesis (Thompson, 1995). IAP (inhibitior of apoptosis) protein family directly inhibits caspase and pro-caspase molecules that function as potential modulators of the terminal effect phase of cell death and survival (Deveraux *et al*, 1997; Roy *et al*, 1997; LaCasse *et al*, 1998). Survivin, a new member of the IAP family, was recently identified to be a novel antiapoptotic gene (Ambrosini *et al*, 1997; Adida *et al*, 1998; Tamm *et al*, 1998). It inhibits apoptosis by binding specifically to the terminal effected cell death proteases, caspase-3 and -7 *in vitro*, thereby inhibiting caspase activity and apoptosis in cells exposed to diverse apoptotic stimuli (Tamm *et al*, 1998). Survivin expression was reported in several apoptosis related foetal tissues, including lung, liver, heart, and gastrointestinal tract (Adida *et al*, 1998), but not in differentiated tissues except for placenta and thymus (Ambrosini *et al*, 1997). Several reports have noticed elevated levels of survivin in tumour tissues when compared to normal tissue (Ambrosini *et al*, 1997; Adida *et al*, 1998; Tamm *et al*, 1998). The role of survivin in gastric carcinogenesis is, however, largely unknown.

Increased cyclo-oxygenase-2 (COX-2) expression has been demonstrated in the process of gastric carcinogenesis (Ristimaki *et al*, 1997; Sung *et al*, 2000; Leung *et al*, 2001). Considerable evidence suggests that the increase in tumorigenic potential of COX-2 expressing cells is related to the resistance to apoptosis (Tsujii and DuBois, 1995). Although the mechanism underlying COX-2-mediated inhibition of apoptosis is largely unknown, previous studies revealed that COX-2 overexpression in intestinal epithelial cells increased level of IAP (Tsujii and DuBois, 1995). Furthermore, prostaglandin E2, the major product of COX-2, has been shown to increase *bcl-2* expression, another suppressor of apoptosis, in human colon cancer cells (Sheng *et al*, 1998). In contrast, treatment with selective COX-2 inhibitors down-regulates *Bcl-2* expression and induces apoptosis (Tsujii and DuBois, 1995). All of these findings indicate that COX-2 is involved in the inhibition of apoptosis signalling by interaction with IAP. In contrast to *bcl-2*, survivin is unique in that it is undetectable in normal adult tissues, but abundantly expressed in transformed cells and a variety of human cancers. Little, however, is known the interaction between COX-2 and survivin.

In the present study, we sought to examine survivin expression in gastric cancer and in first degree-relatives of gastric cancer patients in order to elucidate the role of survivin in the process of gastric carcinogenesis. In addition, we studied the correlation between survivin expression and COX-2 activity in cancerous and non-cancerous gastric tissues.

## MATERIALS AND METHODS

### Cancer cell line

The human gastric cancer cell lines KATO III, MKN45, MKN28, AGS and N87 were obtained from Riken Cell Bank (Tsukuba, Japan) and American Type Culture Collection (Rockville, MD, USA). All cell lines, with the exception of Kato III, were maintained in RPMI 1640 medium (Gibco BRL, Gaithersburg, MD, USA) supplemented with 10% foetal bovine serum (Gibco BRL). Kato III was cultured in 80% RPMI 1640 medium and 20% foetal bovine serum.

### Gastric tissue samples

Fifty patients undergoing surgery for primary gastric cancer were examined. There were 33 males and 17 females with a mean age of 62.2 years (range from 36–83). Paired gastric tumours and adjacent normal gastric mucosa were obtained from each patient at the time of surgery. The samples were immediately frozen in liquid nitrogen and were stored at −80°C. The remaining tissue specimens were fixed in 10% formalin and embedded in paraffin for routine histological examination and immunohistochemical analysis. Tumours were reviewed and classified into intestinal or diffuse types according to Lauren, and the disease was staged according to the UICC/AJCC TNM classification (Sobin and Wittekind, 1997). In addition, 30 first-degree relatives of gastric cancer patients and 20 normal control subjects without a family history of gastric cancer were examined. Gastric biopsy specimens were obtained from the gastric antrum and corpus of these patients. Tissues were processed as mentioned above. The severity of gastritis and *H. pylori* colonization of the non-tumorous gastric mucosa was classified according to the updated Sydney system (Dixon *et al*, 1996). *H. pylori* infection was documented by histology and rapid urease test. This study was approved by the Ethics Committee of Medical Faculty of the Otto-von-Guericke University, Magdeburg and informed consent had been obtained from each subjects.

### RT–PCR and sequencing

Gastric tissue specimens were homogenized with an ultrasound homogenizer. Total RNA was extracted by using RNA Tri Reagents (CINNA/MRC, Cincinnati, Ohio, USA) according to the manufacturer's protocol. One-μg of total RNA was reverse transcribed into cDNA by using dNTPs (1 mM), 5X reverse transcription buffer (500 mM Tris-HCl pH 8.3, 250 mM KCl, 50 mM MgCl2 and 50 mM DTT), 16 units RNasin, and 2.5 units of AMV reverse transcriptase (Gibco BRL, Life Technologies).

**Figure 1:**
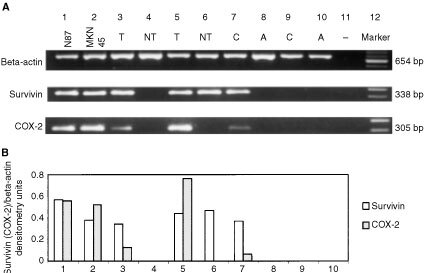
(**A**) Expression of survivin and COX-2 mRNA in gastric cancer cell lines (lane 1: NCI-N87; lane 2: MKN45), gastric cancer tissues (T) and paired non-tumour tissues (NT) (lanes 3–6), corpus (C) and antrum (A) biopsies of first-degree relatives (lanes 7–8) and non-cancer control (lanes 9–10). The 338 bp human survivin-specific sequence, 305 bp human COX-2-specific sequence and a 654 bp β-actin sequence were amplified from cDNA of gastric cancer cell lines and gastric tissues, separated by agarose gel electrophoresis and visualised by ethidium bromide staining. (**B**) Densitometry of survivin and COX-2 transcripts, standardised to β-actin, for the conditions listed above in **A**.

For PCR, the primer sequences and expected product sizes were as follows: survivin, (forward) 5′-GGACCACCGCATCTCTACAT-3′ and (reverse) 5′-GCACTTTCTTCGCAGTTTCC-3′, 338 base pairs (bp); COX-2, (forward) 5′-AGATCATCTCTGCCTGAGTATCTT 3′, (reverse) 5′-TTCAAATGAGATTGTGGGAAAAT-3′, 305 bp; and β-actin, (forward) 5′-TGACGGGGTCACCCACACTGTGCCCATCTA-3′, (reverse) 5′-CTAGAAGCATTTGCGGTGGACGATGGAGGG-3′, 654 bp. The reaction was performed at 95°C for 1.5 min, and followed by 35 cycles of denaturating at 95°C for 24 s, annealing at 58°C for 48 s and extension at 72°C for 1 min. The PCR products were separated on 1.5% agarose gel and saved as digital images (Uvigrab; UVItec, Cambridge, UK) (Figure 1

). The sequences of the PCR products were confirmed by automated sequencing (ABI Prism 310 Genetic Analyzer; Perkin Elmer, Branchburg, NJ, USA).

### Immunohistochemical staining for survivin and COX-2

Expression of survivin and COX-2 protein was examined by avidin-biotin complex (ABC) immunoperoxidase method. Deparaffinized sections were treated with 3% hydrogen peroxide to block endogenous peroxidase activity. After blocking with 5% normal serum for 20 min, the primary antibody was applied and incubated overnight at 4°C for survivin (1 : 200 dilution; #500-201, Novus Biologicals, Littleton, CO, USA) and at room temperature for 2 h for COX-2 (1 : 100 dilution; #SC-1745, Santa Cruz Biotechnology, Santa Cruz, CA, USA) respectively. After rinsing, the biotinylated secondary antibody and ABComplex/HRP (Dako A/S, Denmark) were applied. Peroxidase activity was visualized by applying the diaminobenzidine chromogen. The sections were then counter-stained with haematoxylin. PBS alone without primary antibody was used as negative controls of immunostaining.

Two independent investigators evaluated the immunohistochemical staining without knowledge of the clinico-pathological features of the tumours. For survivin and COX-2, staining in the nucleus and cytoplasm was evaluated by scanning of the whole section and counting more than 1000 representative cells. A semi-quantitative scoring system was used: 0=<5% of cells with expression; 1=5–25%; 2=25–50%; 3=50–75%; and 4=>75% expression. Cases with weighted scores <1 were defined as negative, and were otherwise defined as positive.

### Western blot

**Figure 3:**
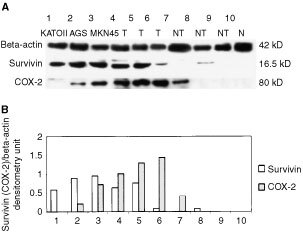
(**A**) Representative samples of Western blotting for survivin and COX-2 proteins in human gastric cancer cell lines (KatoIII, AGS and MKN45), gastric cancer tissues (T), paired non-tumour tissues (NT) and control gastric mucosa (N). The upper panel represents the β-actin levels as the internal control. (**B**) Densitometry of survivin and COX-2 proteins, standardised to β-actin, for the conditions listed above in (A).

Protein was extracted as described previously (Leung *et al*, 2000a). Gastric tissues and cell lines were homogenized in Tris-HCl (pH 7.4) buffer containing 0.5% Triton X-100 and protease inhibitor cocktail (Roche, Indiapolis, IN, USA). Protein concentrations were measured by the method of Bradford (Bio-Rad). Sixty micrograms of protein was loaded per lane, separated by 10% SDS-polyacrylamide gel electrophoresis under reducing conditions, and transferred onto equilibrated polyvinylidene difluoride membrane (Amersham) by electroblotting. Membranes were blocked by 5% non-fat dry milk and then incubated with an antibody against survivin (dilution 1 : 1000) and COX-2 respectively for 3 h. After secondary antibody incubation, survivin and COX-2 was detected by enhanced chemiluminescence method (Pierce, Rockford, IL, USA) (Figure 3

).

### *In situ* DNA nick end labelling (TUNEL)

**Figure 2:**
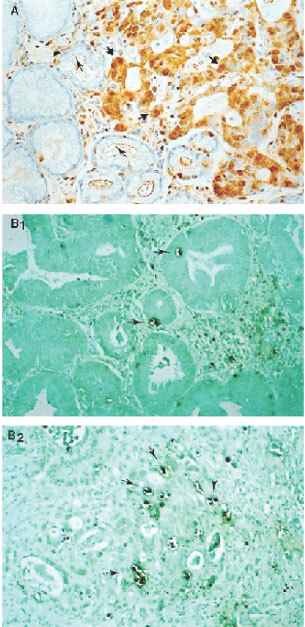
(**A**) Staining of survivin was mainly restricted to tumour cells (short arrow) but was rarely in the adjacent normal mucosa in this sample (long arrow, ×250). (**B**) Apoptotic nuclei as revealed by TUNEL in survivin-positive tumour (long arrow, B1) and in survivin-negative tumour (long arrow, B2, ×250).

Apoptosis was determined by the terminal deoxynucleotidyl transferase (TdT)-mediated deoxyuridine triphosphate nick-end labelling (TUNEL) technique (ApopTag; Intergen, Purchase, NY, USA) as described previously (Leung *et al*, 2000b). Nuclei with clear brown staining were regarded as positive (Figure 2B

). The apoptosis index was calculated as the percentage of TUNEL-positive nuclei after counting more than 1000 epithelial cells.

### Statistical analysis

Statistical analysis was performed using the Statistical Package for the Social Science program (SPSS, version 9.0). The correlation between survivin expressions and various clinicopathological features of tumours was analysed by using either the χ^2^ test or Student's *t*-test. The association between survivin and COX-2 was analysed by Spearman's rank correlation analysis. Differences in apoptosis index were analysed by the independent Wilcoxon test. A two-side *P* value of less than 0.05 was considered statistically significant.

## RESULTS

### Expression of survivin mRNA in gastric cancer and in cancer cell lines

Survivin mRNA expression was undetectable in the normal gastric mucosa by RT–PCR. By contrast, all five cancer cell lines, exhibited survivin expression. Among the 50 gastric cancer samples, survivin mRNA was detected in 34 (68%) cases whereas 11 (22%) of the adjacent non-tumour samples demonstrated survivin expression (*P*<0.001). Survivin mRNA was, however, not detected in the neighbouring normal tissues when the corresponding cancer tissues were negative (Figure 1, Table 1

**Table 1:**
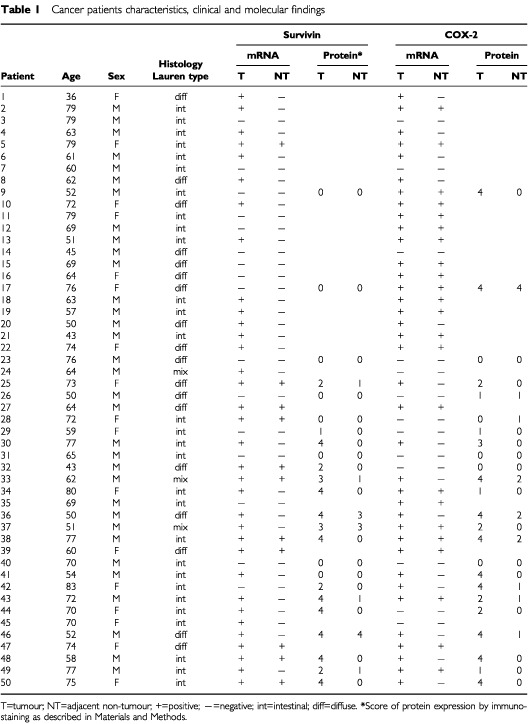
Cancer patients characteristics, clinical and molecular findings

). There was no association between survivin expression and clinical-pathological parameters of the gastric cancers including patients' demographic data, tumour staging, presence of lymph node metastasis and tumour subtypes (Table 2

**Table 2:**
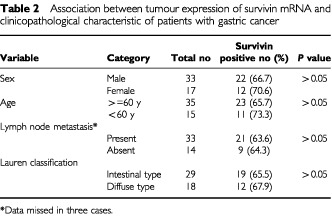
Association between tumour expression of survivin mRNA and clinicopathological characteristic of patients with gastric cancer

).

### Survivin protein expression in gastric cancer and cancer cell lines

Survivin protein expression was studied by Western blot and immunohistochemistry in 24 randomly selected gastric cancers and the corresponding adjacent normal gastric tissues as well as in the gastric mucosa of 20 non-cancer individuals. In keeping with the findings by RT–PCR, there was no positive staining in the normal gastric mucosa tissues from non-cancer patients. In contrast, survivin was strongly expressed in gastric cancer tissues (Figure 2). Staining for survivin was observed primarily in the nuclei but was also weakly present in the cytoplasm. Fifteen out of 24 cases (62.5%) of gastric cancers were positive while three (12.5%) of the adjacent non-cancer mucosa exhibited survivin protein (*P*=0.001) (Figure 2A). Expression of survivin in gastric cancer and cancer cell lines was further confirmed by immunoblot which revealed a 16.5 kDa band protein (Figure 3). Strong expression of survivin protein was demonstrated in 14 of the 24 (58.3%) gastric cancer tissues and in all five gastric cancer cell lines. Weak expression was detected in the adjacent normal mucosa whereas survivin protein was undetectable in normal controls. The presence of the respective mRNA moiety was more frequent than the respective protein, due to the diverse sensitivity of the methods used for analysis. However, for survivin a consistent correlation between the survivin protein and mRNA levels was observed.

### Survivin expression in first-degree relatives

A total of 30 first-degree relatives were examined for survivin mRNA expression in the gastric corpus and antrum. Increased survivin expression was seen in seven corpus biopsies (23%) and in five antral biopsies (17%) (Table 3

**Table 3:**
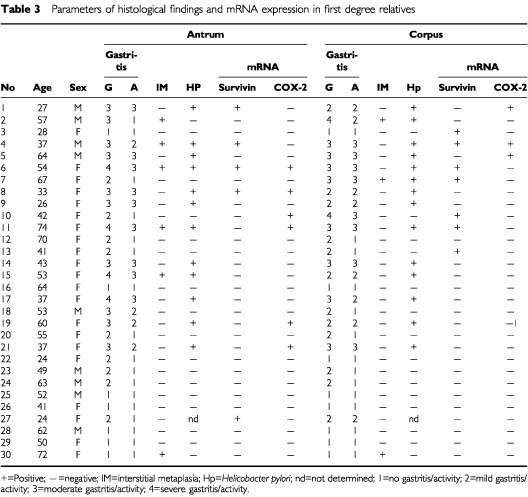
Parameters of histological findings and mRNA expression in first degree relatives

). When compared to normal gastric tissues from the 20 control subjects, survivin expression was more frequently detected in both the corpus (*P*=0.02) and the antrum (*P*=0.046) of first-degree relatives. Among the 60 specimens obtained from the corpus and antrum of first-degree relatives, six were classified as severe gastritis, 18 as moderate gastritis, 21 as mild gastritis and 15 as normal. Survivin expression was more often increased in gastric biopsies with moderate and severe gastritis (9/24, 37.5%) than in samples with mild or no gastritis (3/36, 8.3%; *P*=0.009). In keeping with the findings by RT–PCR, staining for survivin was observed in gastric epithelial cells in two out of 10 first degree relatives.

### Survivin expression and *H. pylori* infection

First-degree relatives and control subjects with proper diagnosis of *H. pylori* infection were studied for the relationship between survivin mRNA expression and *H. pylori* infection. In total, 88 gastric tissue samples from relatives and normal control subjects were analysed. Eight of 30 (26.7%) specimens with *H. pylori* infection showed increased survivin expression, whereas three out of 58 (5.2%) specimens without *H. pylori* infection had survivin expression (*P*=0.006).

### Correlation between survivin expression and apoptosis

The relationship between survivin expression and apoptosis index of gastric tumours was studied. In gastric tumours with survivin expression, the mean apoptosis index was 1.07% (SD=0.54), which was significantly lower than survivin-negative tumours (1.80%, SD=1.62; *P*=0.02) (Figure 2B).

### Correlation between survivin and COX-2 expression

As shown in Table 1, increased COX-2 mRNA expression was detected in 37 of 50 (74%) gastric cancer and 23 (46%) adjacent non-tumour tissues. By immunohistochemistry, COX-2 protein expression was found in 15 of 24 (62.5%) cancers and four (16.7%) adjacent normal specimens. The correlation between survivin and COX-2 expression was evaluated in the same tissue specimens of each individual patient by RT–PCR and by immunohistochemistry. There was a significant correlation at per case level between survivin and COX-2 mRNA expression in gastric tumour samples (*r*=0.523; *P*=0.001). In agreement with mRNA expression, a similar correlation was demonstrated by parallel immunostaining of gastric cancer specimens (*r*=0.420, *P*=0.041). However, a similar correlation could not be demonstrated in adjacent non-tumour tissues and in gastric biopsies obtained from first-degree relatives.

## DISCUSSION

Development of gastric cancer, like many other malignancies, is a multi-step process involving the accumulation of mutations and changes in cell cycle regulatory mechanisms. The detection of these alterations in the early stage of cancer development may shed new light into the gastric carcinogenesis process. Our previous studies showed that these changes are not merely found in gastric cancer cells but also in adjacent non-tumour tissues as well as in first-degree family members of cancer patients (Ebert *et al*, 2000; Yu *et al*, 2000a,b). In this study, survivin mRNA expression was detected in the majority of gastric cancers and in all cancer cell lines. In this regard, two recent studies showed that 35–82% of gastric cancers express survivin protein by using immunohistochemistry (Lu *et al*, 1998; Okata *et al*, 2001). In contrast to previous studies, we also examined survivin expression at the RNA and protein levels. In this study, we demonstrated that survivin was not detected in the gastric mucosa from normal control subjects whereas the adjacent non-tumour gastric mucosa of survivin-positive tumours occasionally expressed survivin. This finding is in keeping with those found in colorectal and oesophageal cancer where survivin expression was demonstrated in adjacent normal tissues (Sarela *et al*, 2000; Kato *et al*, 2001).

Past epidemiological studies revealed that first-degree relatives have an approximately three-fold increase in risk of developing gastric carcinoma, suggesting the existence of a genetic susceptibility to cancer (Zanghieri *et al*, 1990; La Vecchia *et al*, 1992). In this study, we observed an increased survivin mRNA expression in the gastric mucosa of first-degree relatives but not in normal control. Thus, survivin may play an important role in the early stage of development of gastric cancer within family members. One of the plausible explanations for these findings may be related to *H. pylori* infection. *H. pylori* infection is considered to be an important triggering factor for gastric carcinogenesis (Correa, 1992). Notably, our present study demonstrates that survivin expression correlates with the severity of gastritis and *H. pylori* infection in non-cancer patients. On the other hand, this appears to contradict with the observation that chronic gastritis and *H. pylori* infection is associated with increased epithelial cell apoptosis (Attallah *et al*, 1996; Moss *et al*, 1996; Wagner *et al*, 1997; Leung *et al*, 2000b). We speculated that the increased survivin expression in these tissues may be secondary to the pro-apoptotic stimuli induced by *H. pylori*. Up-regulation of survivin, an anti-apoptosis protein, then offers an advantage to a rapidly growing tumour by slowing down the cell loss rate and ultimately leading to neoplastic transformation. This study may offer a further possible biological link between chronic *H. pylori* infection and gastric cancer development.

In this study, gastric cancers with survivin expression had a reduced apoptosis index, which is in keeping with the previous report by Lu *et al* (1998). Additionally, these investigators demonstrated an association between survivin and p53 and *bcl-2* expression in gastric cancers. While p53 and *bcl-2* are involved in the modulation of cell progression and viability, the role of other mediators of apoptosis has not been examined yet. Previously, we have demonstrated COX-2 up-regulation in gastric cancer and pre-neoplastic gastric lesions that may play contribute to the carcinogenesis process (Sung *et al*, 2000; Leung *et al*, 2001). Overexpression of COX-2 in intestinal epithelial cells results in inhibition of apoptosis (Tsujii and DuBois, 1995), which is similar to the action of survivin. A key question we have addressed here is whether there is any correlation between COX-2 and survivin expression in gastric tumour. Our results show a strong association between survivin and COX2 expression in gastric cancer. The co-expression of survivin and COX-2 demonstrated in this study is intriguing. Survivin blocks apoptosis by targeting the terminal effector caspase-3 and caspase-7 (Adida *et al*, 1998; Tamm *et al*, 1998) whereas stimulation of ceramide may be one of the molecular basis for the anti-apoptotic effect of COX-2 (Chan *et al*, 1998). Whether there is any link between these intermediate pathways may require further studies. Alternatively, common transcriptional factors may be involved in the co-regulation of survivin and COX-2 gene in gastric cancer.

In summary, we have demonstrated the up-regulation of survivin in gastric cancer and the adjacent non-tumour area as well as in a proportion of first-degree cancer relatives. Expression of survivin in gastric cancer was associated with reduced apoptosis and COX-2 expression. On the other hand, expression of survivin in non-neoplastic tissues is closely associated with *H. pylori* infection and gastric inflammation. Collectively, these findings indicate that inhibition of apoptosis regulated by survivin is important in the pathogenesis of gastric cancer.
